# Supplementary material for: In vitro evolution of an influenza broadly neutralizing antibody is modulated by hemagglutinin receptor specificity
Source: Nat Commun. 2017 May 15;8:15371. doi: 10.1038/ncomms15371 (PMC5440694; doi:10.1038/ncomms15371)
Supplement: Supplementary Information — Supplementary figures, supplementary tables and supplementary references. [file ncomms15371-s1.pdf]

Supplementary Table 1. Concentration of hemagglutinin trimer used for selection.

	H1 Hemagglutinin A/Solomon Islands/3/2006	H3 Hemagglutinin A/Perth/16/2009	H5 Hemagglutinin A/Vietnam/1203/2004
Round 1	30 nM	30 nM	30 nM
Round 2	5 nM	2 nM	30 nM
Round 3	0.5 nM	0.15 nM	30 nM

Supplementary Table 2. Barcode sequences for Illumina sequencing.

Sample	Barcode
Input	CGTGAT
H1 HA Round 1	ACATCG
H1 HA Round 2	GCCTAA
H1 HA Round 3	TGGTCA
H3 HA Round 1	CACTGT
H3 HA Round 2	ATTGCC
H3 HA Round 3	GATCTG
H5 HA Round 1	CTGATC
H5 HA Round 2	AAGCTA
H5 HA Round 3	GTAGCC

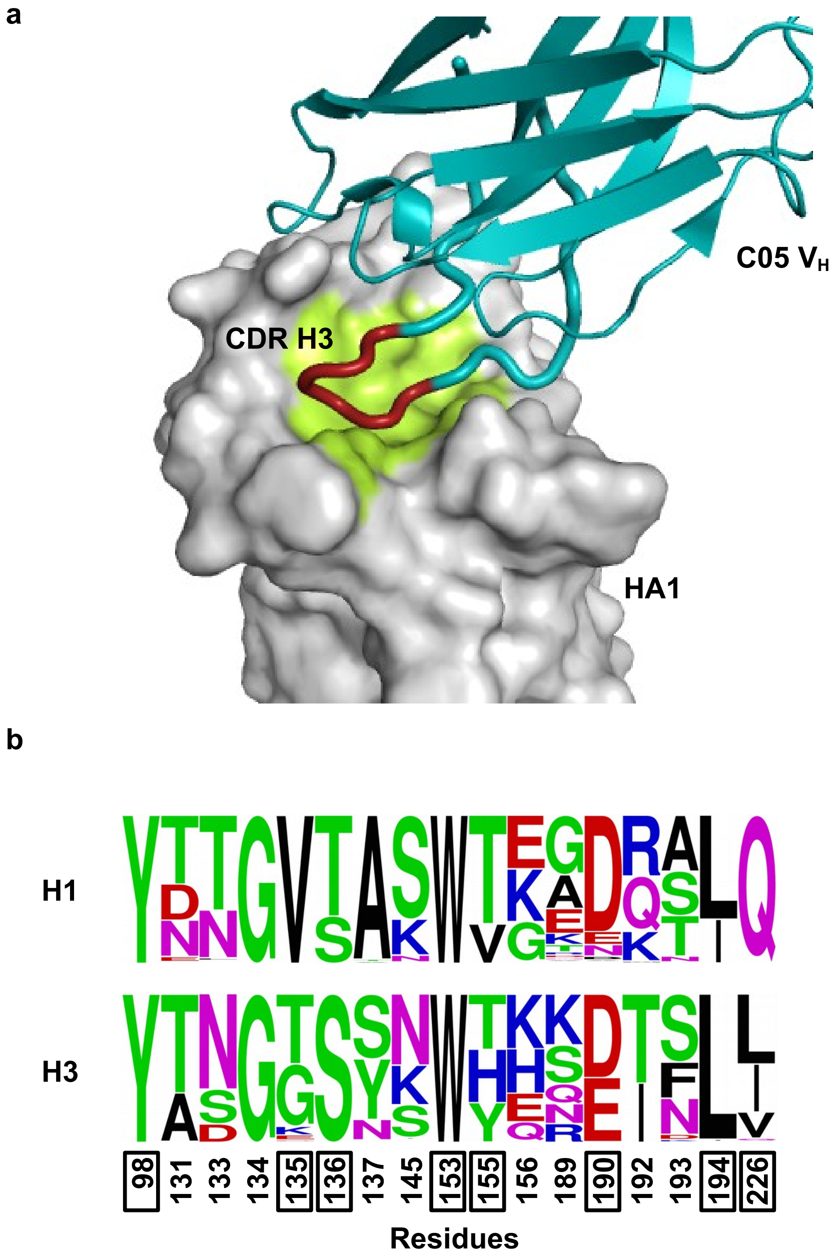

Supplementary Figure 1. Interaction between C05 and HA. (A) The C05 heavy chain variable region is colored in cyan with the six residues of interest colored in red. Influenza hemagglutinin (HA) is colored in grey (PDB: 4FP8)[1], with the receptor binding site in lime. **(B)** The sequence logos represent the natural sequence variation of C05 epitope in H1 or H3 human influenza viruses. HA amino-acid sequences were downloaded from the Influenza Research Database (https://www.fludb.org/)[2], aligned by MAFFT[3] and visualized by WebLogo (http://weblogo.berkeley.edu/logo.cgi)[4]. For each subtype, we randomly sampled at most 10 strains from each year for this analysis. A total of 326 strains were used for H1 subtype and 419 strains were used for H3 subtype. Residues that interact with the six residues of interest are boxed.

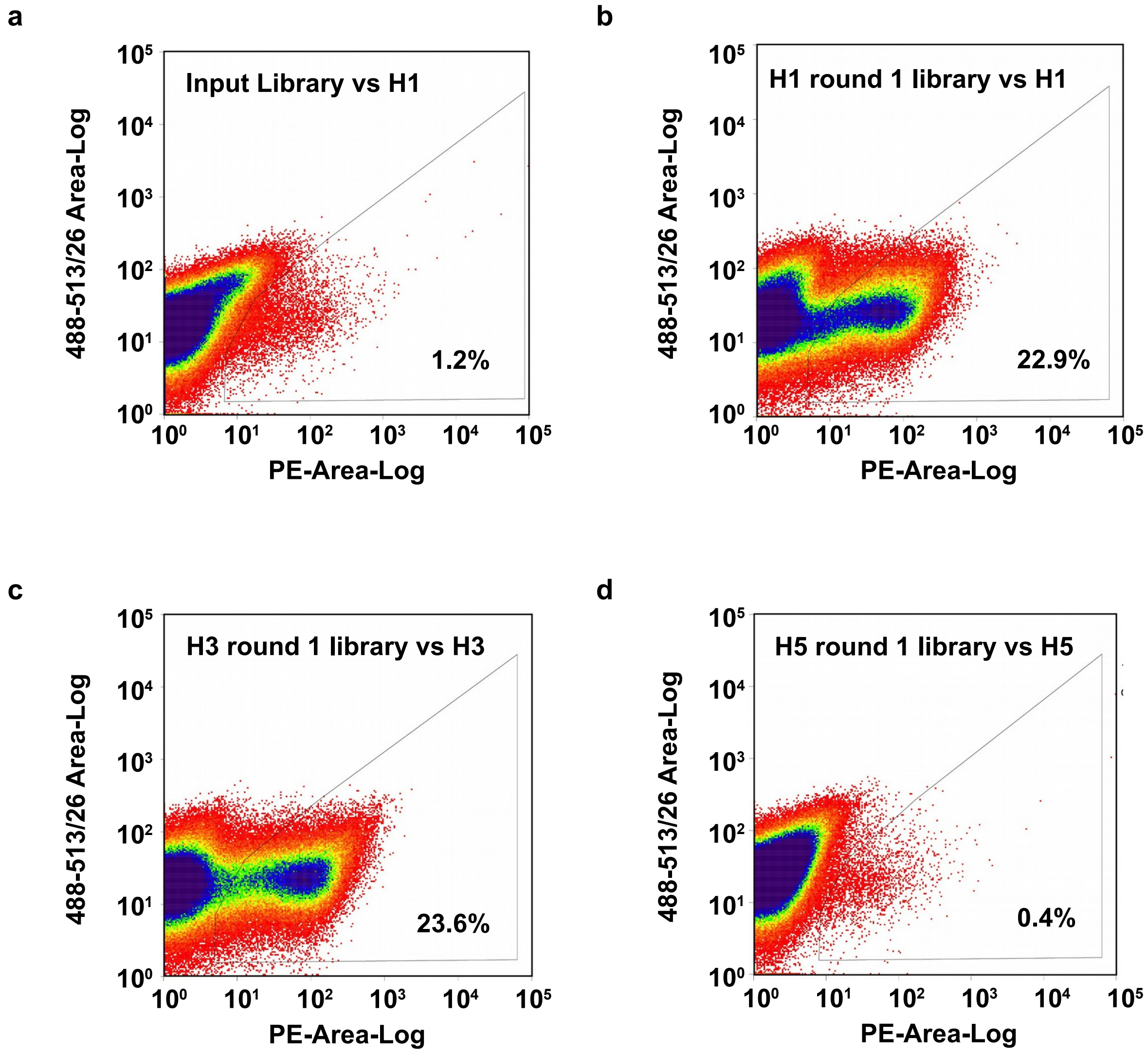

Supplementary Figure 2. Fluorescence-activated cell sorting of C05 Fab yeast display library. Representative results from fluorescence-activated cell sorting (FACS) are shown. **(a)** Input library against H1 HA (A/Solomon Islands/3/2006). **(b)** Post-round 1 H1 selection library against H1 HA (A/Solomon Islands/3/2006). **(c)** Post-round 1 H3 selection library against H3 HA (A/Perth/16/2009). **(d)** Post-round 1 H5 selection library against H5 HA (A/Vietnam/1203/2004).

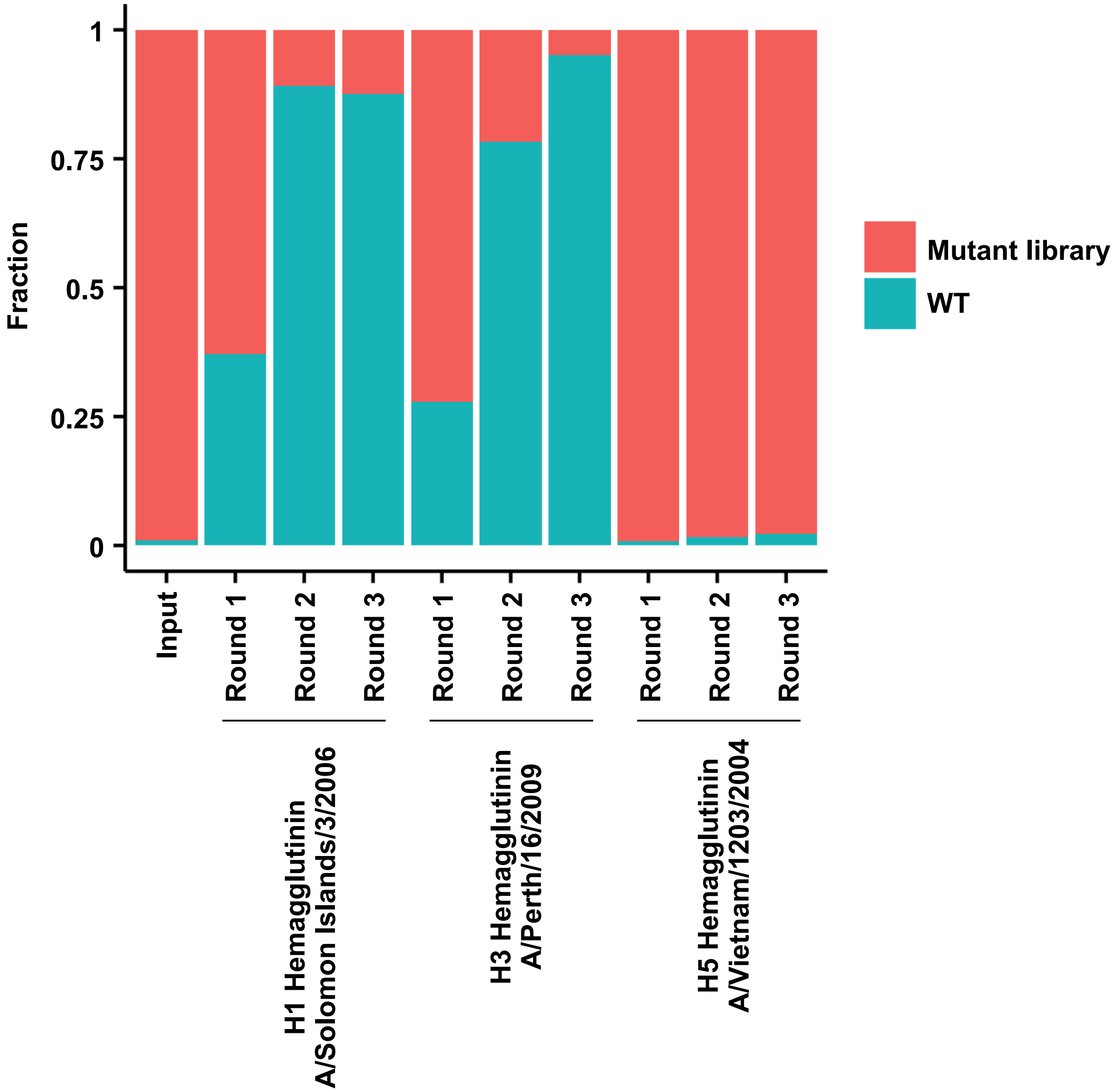

Supplementary Figure 3. Composition of the mutant library. The occurrence frequencies of WT clone and clones belong to the mutant library are shown. WT clone originated from incomplete restriction digestion during the cloning process for mutant library construction. Nucleotide sequence of WT could be distinguished from those clones in the mutant library based on codon usage because the third positions of codons for the region of interest were either G or T in the mutant library due to the codon randomization scheme. However, the third positions of codons in the region of interest in the nucleotide sequence of WT contained nucleotides other than G and T. Consequently, certain clones in the mutant library may encode the same protein sequence as the WT clone, but would have a different nucleotide sequence compared to the WT clone. Those clones, despite encoding the WT protein sequence, would be categorized as belonging to the mutant library during data analysis.

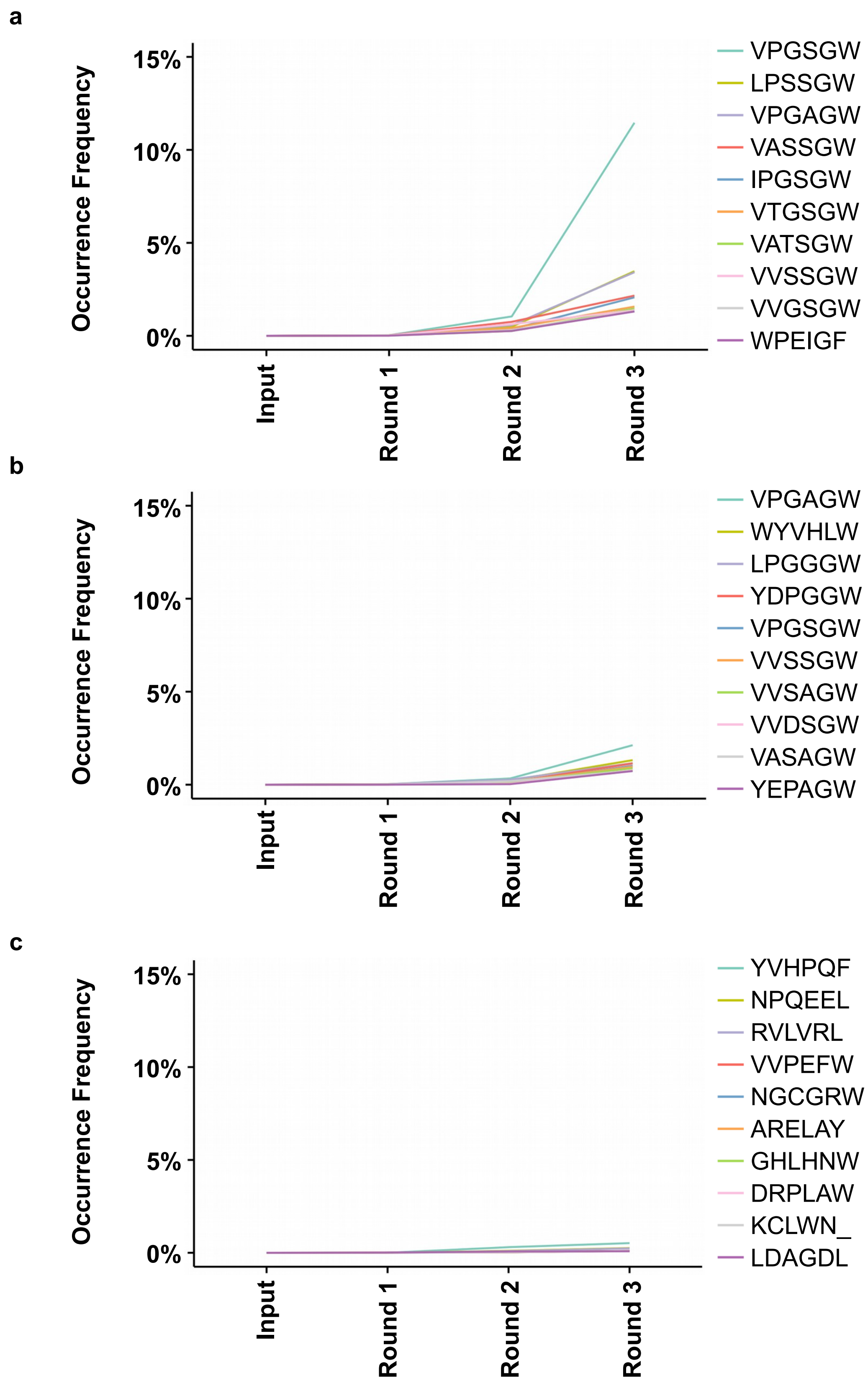

Supplementary Figure 4. Frequencies of top 10 variants after three-round selection. The change in frequencies during the selection process were shown for: **(a)** The top 10 variants in post three-round selection against H1 Hemagglutinin (A/Solomon Islands/3/2006). **(b)** The top 10 variants in post three-round selection against H3 Hemagglutinin (A/Perth/16/2009). **(c)** The top 10 variants in post three-round selection against H5 Hemagglutinin (A/Vietnam/1203/2004).

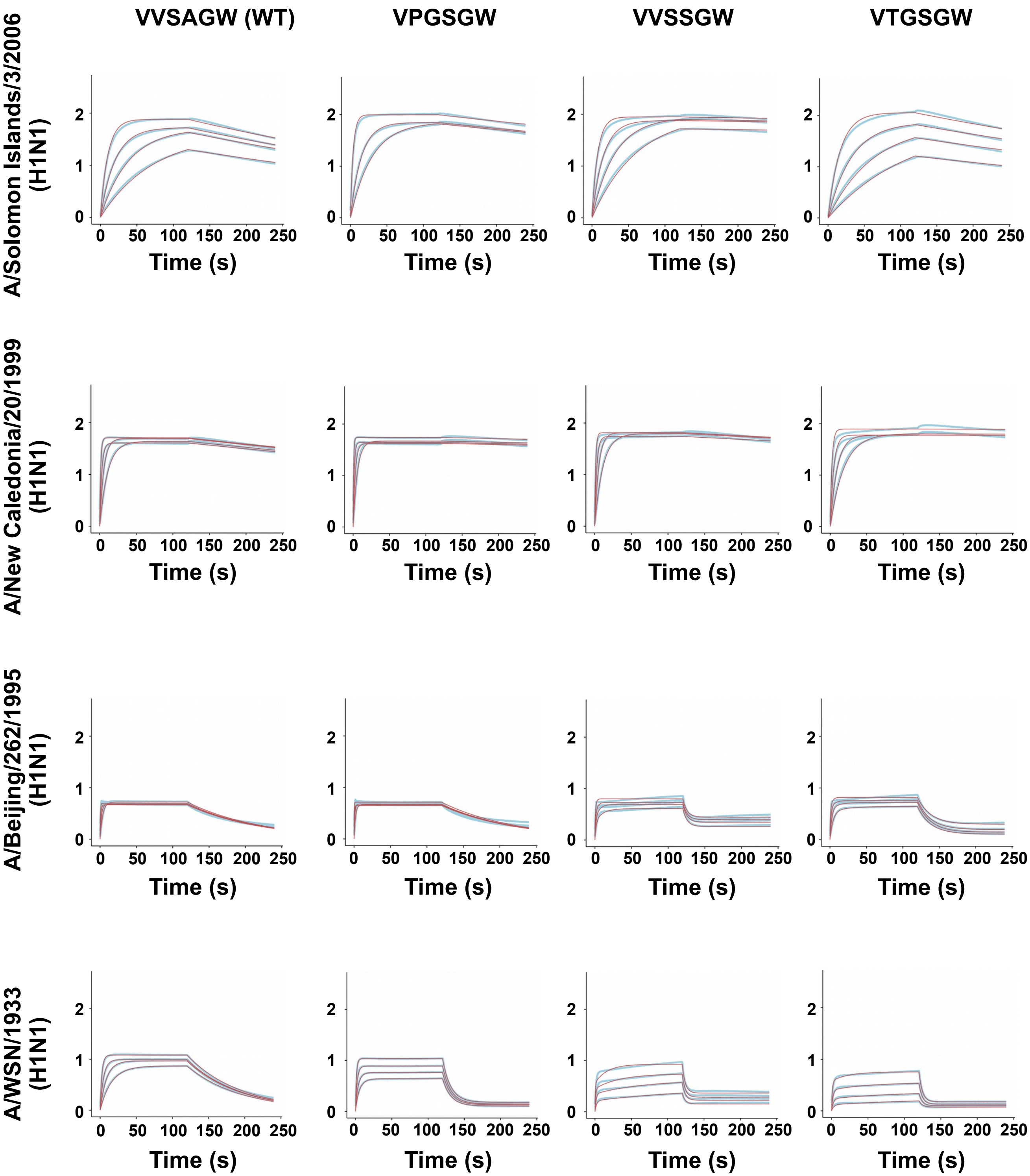

Supplementary Figure 5. Sensorgrams for binding of C05 variants to HAs from different H1 strains. Binding kinetics of C05 WT Fab and three different C05 Fab variants against recombinant HA from different H1 strains were measured by biolayer interferometry (BLI). Y-axis represents the response. Blue lines represent the response curve and red lines represent the best fit model (1:1 binding model or 2:1 heterogeneous ligand model, see Methods). Binding kinetics were measured for three to four concentrations of Fab at 2-fold dilution ranging from 5,000 nM to 625 nM.

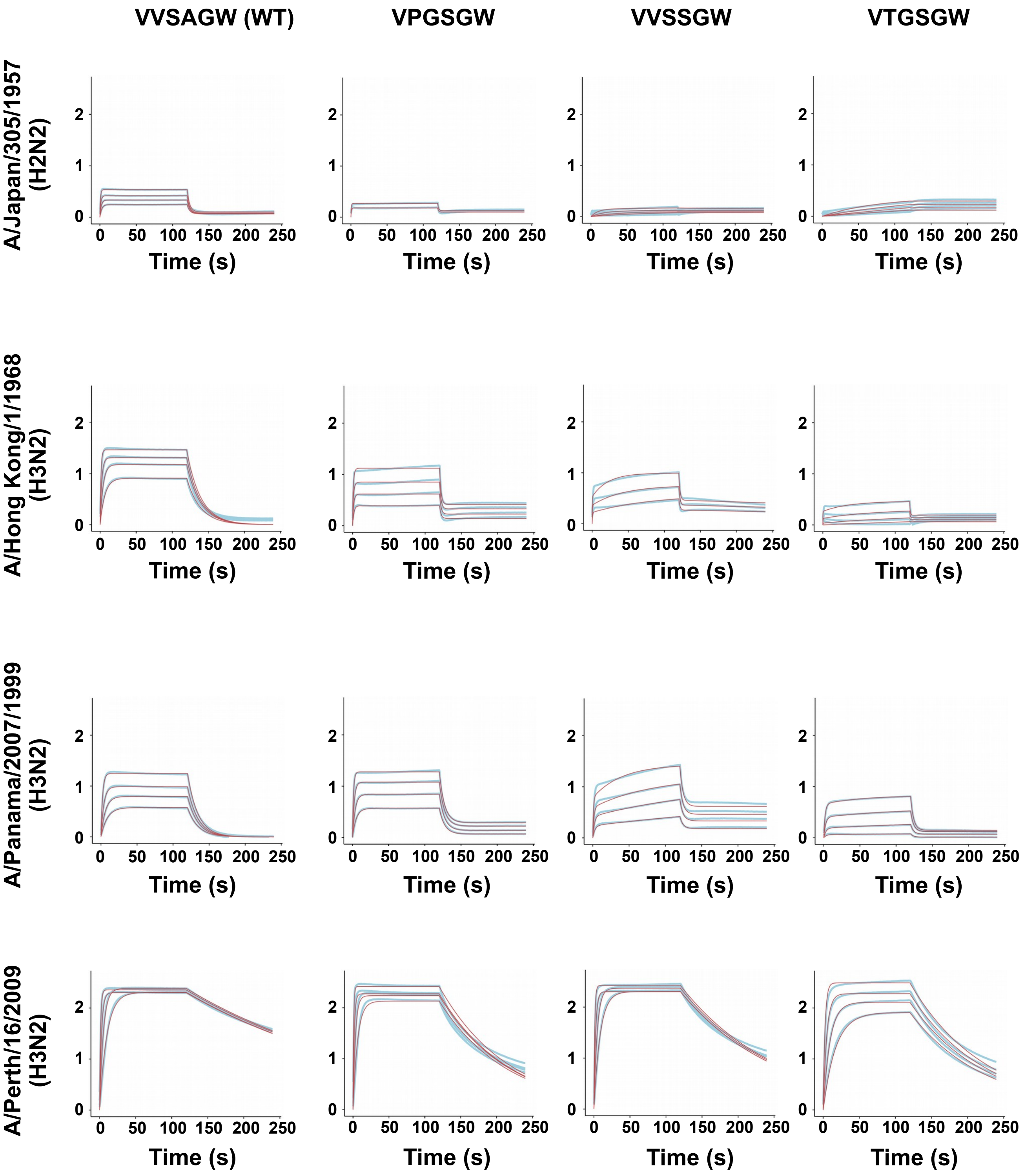

Supplementary Figure 6. Sensorgrams for binding of C05 variants to HAs from different H2 and H3 strains. Binding kinetics of C05 WT Fab and three different C05 Fab variants against recombinant HA from different H2 and H3 strains were measured by biolayer interferometry (BLI). Y-axis represents the response. Blue lines represent the response curve and red lines represent the best fit model (1:1 binding model or 2:1 heterogeneous ligand model, see Methods). Binding kinetics were measured for three to four concentrations of Fab at 2-fold dilution ranging from 5,000 nM to 625 nM.

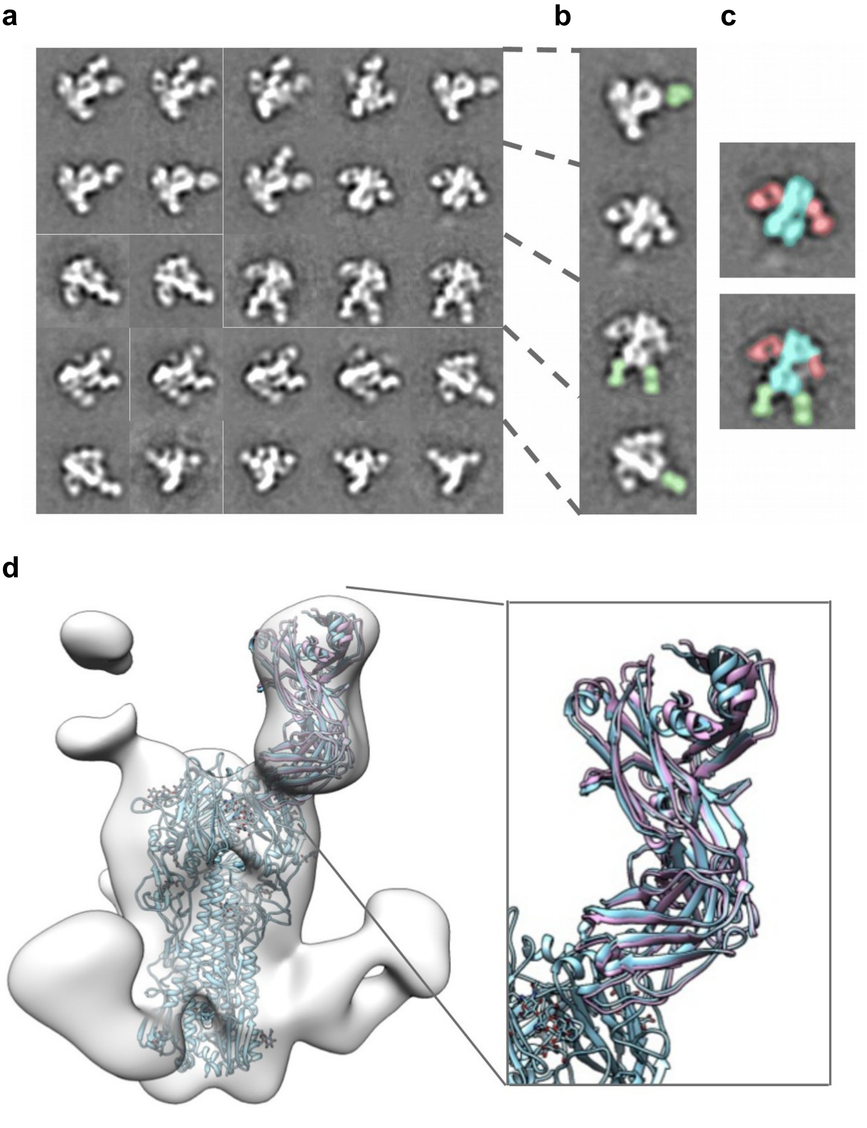

Supplementary Figure 7. Negative-stain electron microscopy reconstruction of VPGSGW and CR9114 Fab in complex with H1 HA trimer. (a) Negative-stain EM 2D classes of VPGSGW and CR9114 Fab in complex with hemagglutinin (HA) trimer from A/Solomon Islands/3/2006 (H1N1). **(b)** VPGSGW is highlighted in green to show its binding to the head region of HA. **(c)** CR9114 Fab is highlighted in red and VPGSGW is highlighted in green to show their binding to the HA stem and HA head respectively. HA is colored in cyan. **(d)** EM negative-stain reconstruction of VPGSGW and CR9114 Fab in complex with H1 HA trimer. Crystal structure of the WT C05 Fab in complex with H3 HA from A/Hong Kong/1/1968 (PDB: 4FQR, blue)[1] and the structure of VPGSGW (in pink) were docked into the asymmetric 3D reconstruction. The low occupancy of VPGSGW was likely due to the complex formation and purification scheme (see Methods).

a

b

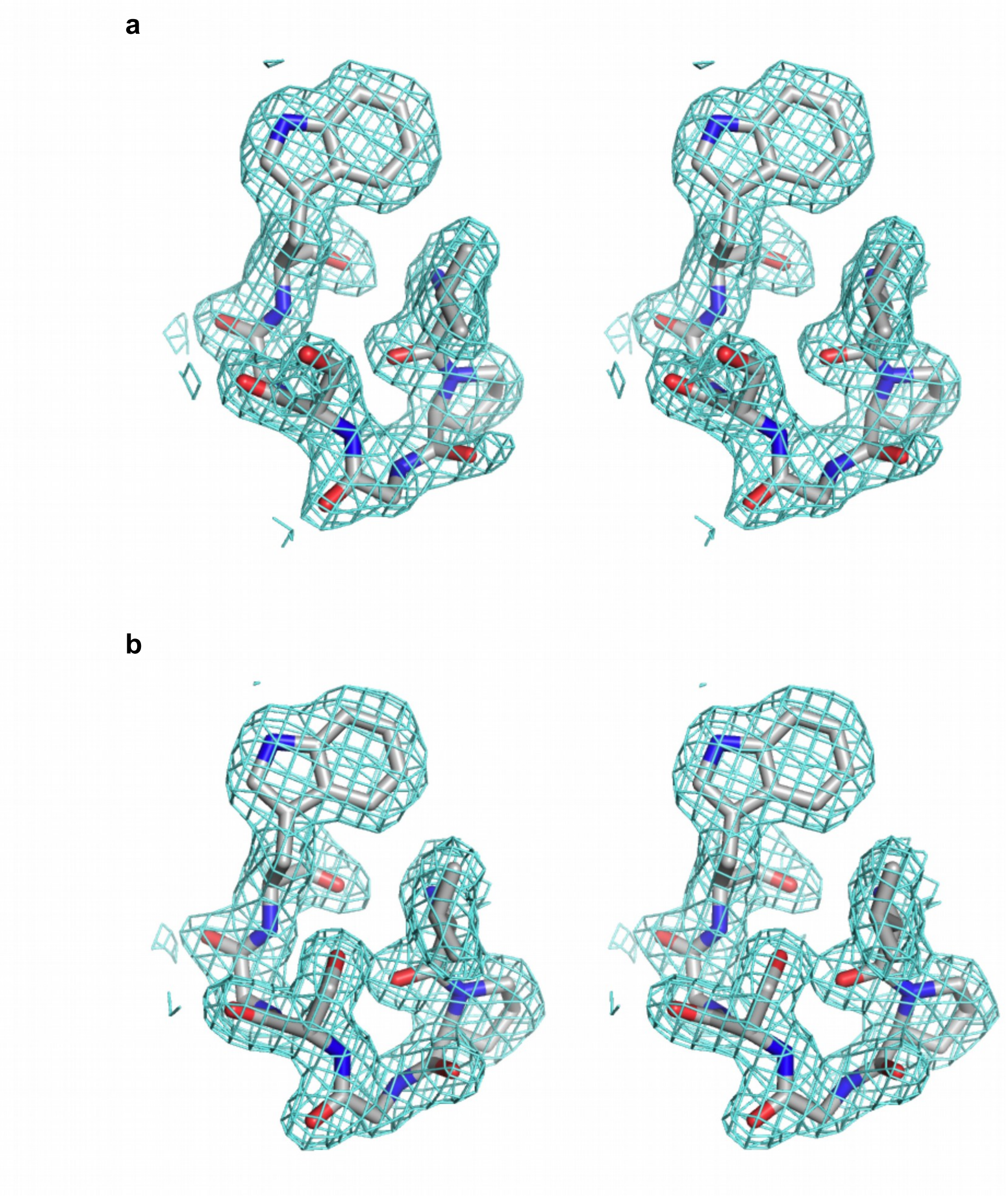

Supplementary Figure 8. Electron density map of VPGSGW. Two VPGSGW-HA1 complexes are present in the asymmetric unit of the crystal structure. Stereo representation of the electron density map of the region of interest in each of the VPGSGW Fabs in the crystal asymmetric unit is shown: **(a)** chain C and **(b)** chain E in the PDB coordinate file (5UMN). The 2Fo-Fc electron density map is represented in a cyan mesh contoured at 1.0 σ with the refined coordinates of the mutated VPGSGW tip of the CDR H3 loop of Fab C05.

a

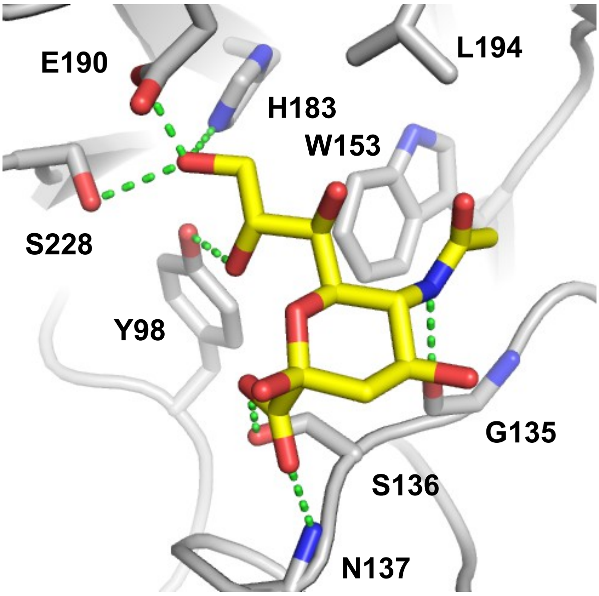

b

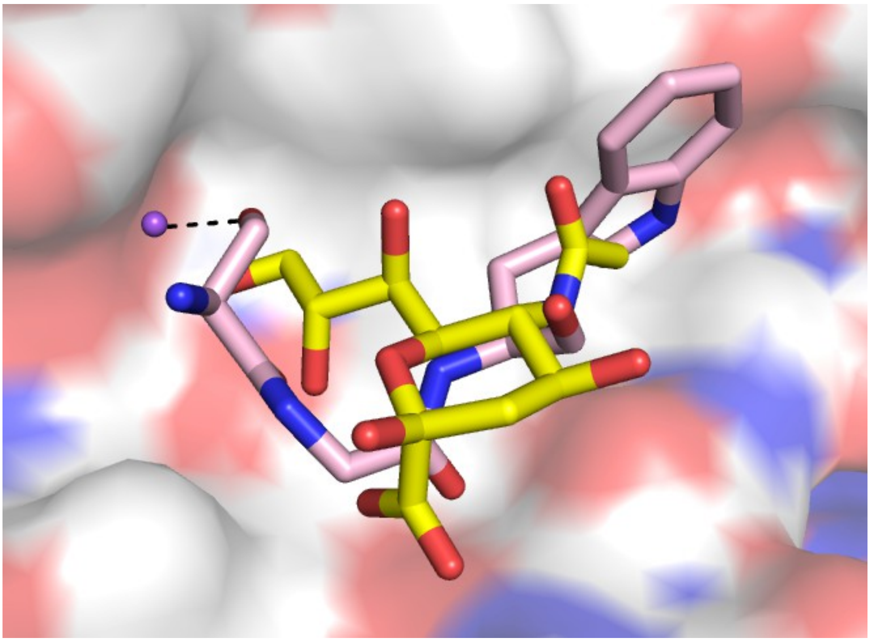

Supplementary Figure 9. The interaction between sialic acid and HA RBS. (a) The interaction between sialic acid (yellow) and influenza A/X-31/1968 hemagglutinin (H3N2) is shown (PDB: 2YPG)[5]. **(b)** Sialic acid (yellow) and part of the six-residue loop from VPGSGW (i.e. S100d, G100e, W100f) are compared in the context of binding to HA RBS. Ion-dipole interaction is represented by the dashed line. A putative sodium ion is represented by the purple sphere.

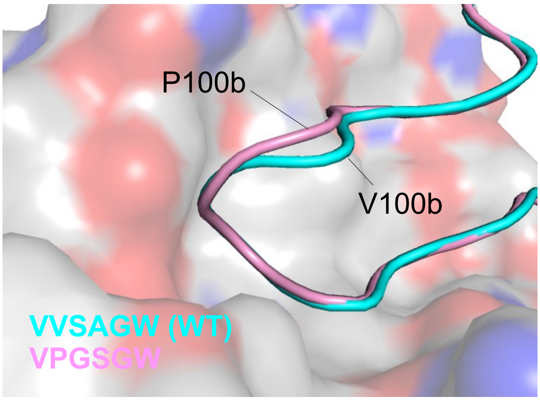

Supplementary Figure 10. Comparison of backbone conformation in WT C05 (VVSAGW) and VPGSGW. The HA RBS is shown by atom types (blue: nitrogen, red: oxygen, grey: carbon). The backbones of the region that inserts into HA RBS is shown for WT C05 (cyan) and VPGSGW (pink). The conformations of their backbones are highly similar, yet exhibit a noticeable difference at residue 100b.

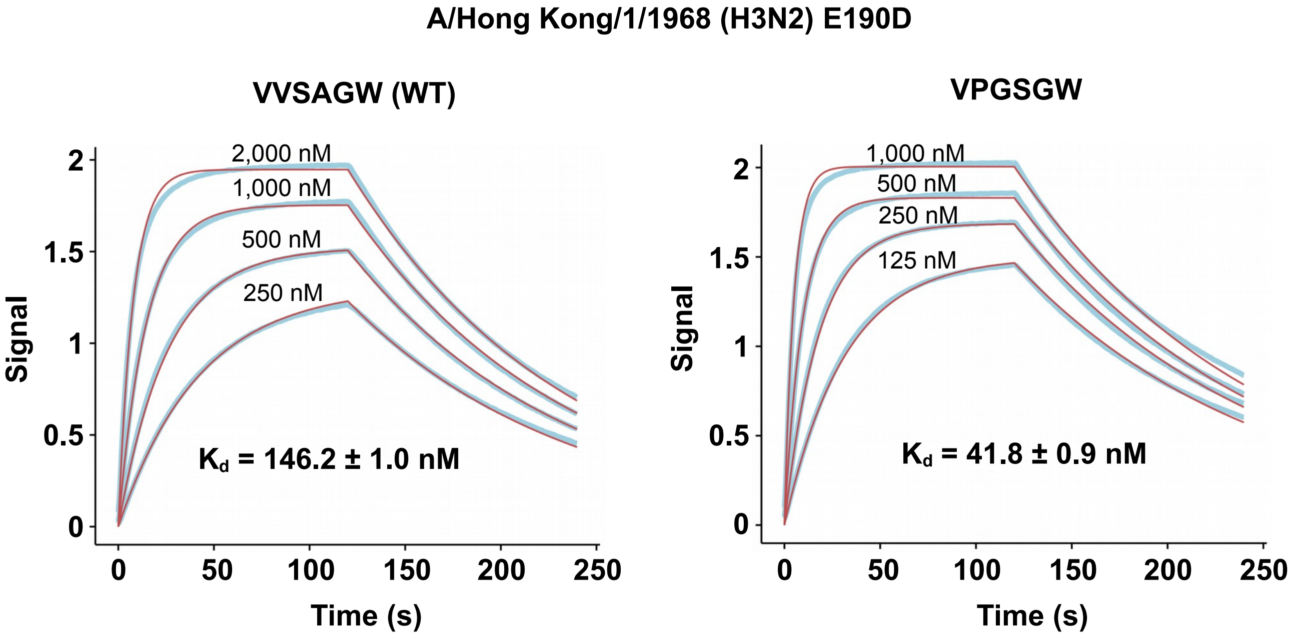

Supplementary Figure 11. Sensorgrams of C05 Fab against E190D HA from A/Hong Kong/1/1968 (H3N2). WT C05 Fab and a C05 variant were purified and tested against immobilized HA from A/Hong Kong/1/1968 (H3N2) that carries the E190D substitution using biolayer interferometry. Blue lines represent the response curve and red lines represent the best fit model (1:1 binding model or 2:1 heterogeneous ligand model, see Methods).

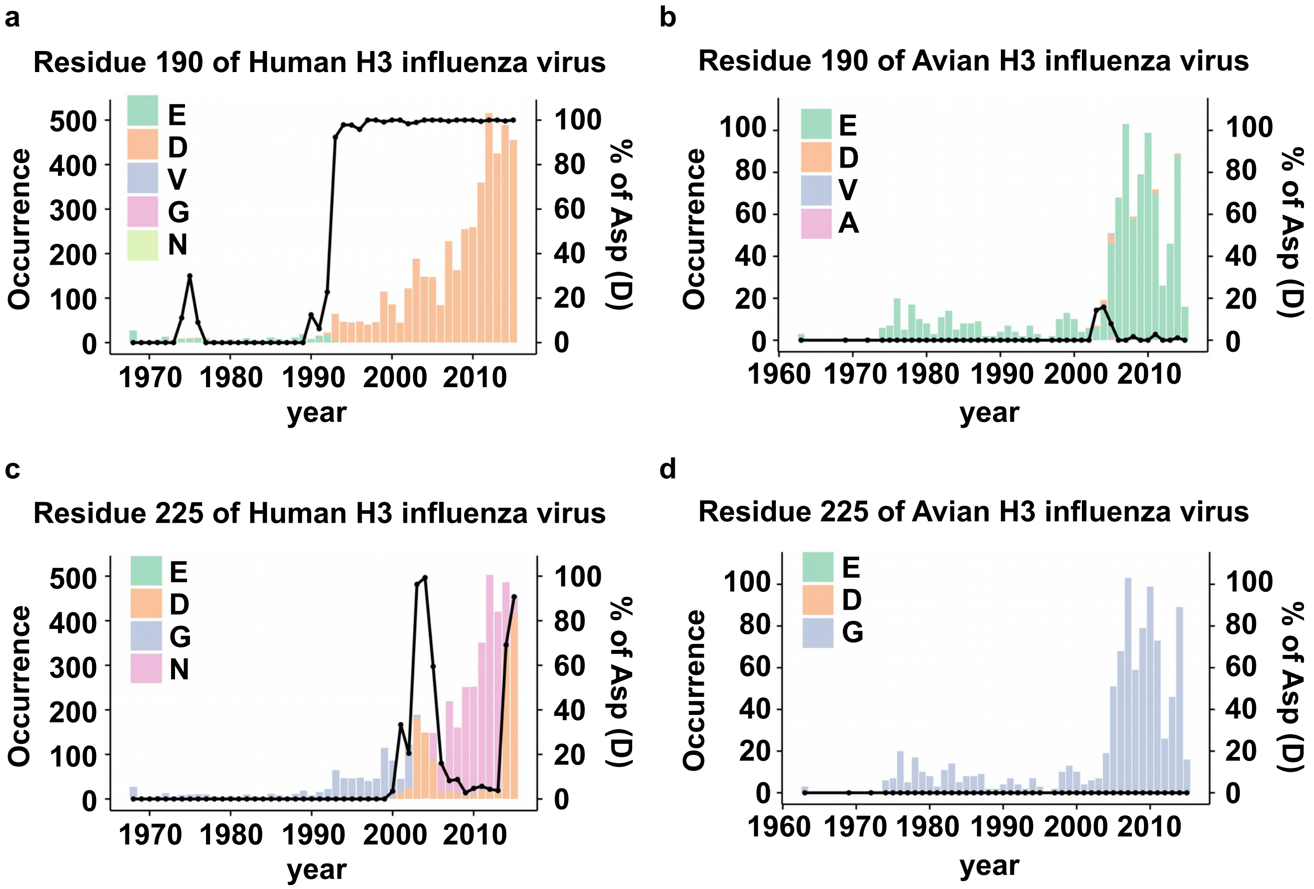

Supplementary Figure 12. Amino-acid identity at residues 190 and 225 of naturally circulating H3 influenza viruses. H3 subtype hemagglutinin (HA) protein sequences were downloaded from the Influenza Research Database (https://www.fludb.org/)[2] and aligned by MAFFT[3]. This set of sequences contained 4,618 isolates from human and 946 isolates from avian. The occurrences of individual amino acids at residues 190 and 225 at the indicated year are shown as a stacked bar plot (y-axis on the left). The occurrence of some minor variants may be too low such that their presence is not visually detectable at the resolution of these graphs. The black line represents the percentage of sequences that carry an Asp at residue 190 at each year (y-axis on the right). **(a)** Most isolates have an Asp or a Glu at residue 190 of human H3 influenza viruses. Minor variants include Val, Gly and Asn. **(b)** Most isolates have a Glu at residue 190 of avian H3 influenza viruses. Minor variants include Asp, Val, and Ala. **(c)** Most isolates have an Asp, a Gly, or an Asn at residue 225 of human H3 influenza viruses. Glu is a minor variant. **(d)** Most isolates have a Gly at residue 225 of avian H3 influenza viruses. Minor variants include Glu and Asp.

Supplementary References

1. Ekiert DC*, et al.* Cross-neutralization of influenza A viruses mediated by a single antibody loop. *Nature* **489**, 526-532 (2012).

2. Squires RB*, et al.* Influenza research database: an integrated bioinformatics resource for influenza research and surveillance. *Influenza Other Respir Viruses* **6**, 404-416 (2012).

3. Katoh K, Standley DM. MAFFT multiple sequence alignment software version 7: improvements in performance and usability. *Mol Biol Evol* **30**, 772-780 (2013).

4. Crooks GE, Hon G, Chandonia JM, Brenner SE. WebLogo: a sequence logo generator. *Genome Res* **14**, 1188-1190 (2004).

5. Lin YP*, et al.* Evolution of the receptor binding properties of the influenza A(H3N2) hemagglutinin. *Proc Natl Acad Sci U S A* **109**, 21474-21479 (2012).
